# Communication experiences of healthcare students whilst managing adults with communication disorders

**DOI:** 10.4102/sajcd.v69i1.870

**Published:** 2022-05-31

**Authors:** Vrinda Rathiram, Lauren O. Neilson, Azraa Syed Kassim, Winnie T. Mokone, Caitlin C. Green

**Affiliations:** Department of Speech-Language Pathology, Faculty of Health Sciences, University of KwaZulu-Natal, Durban, South Africa

**Keywords:** communication experiences, healthcare students, adults, communication disorders, challenges, strategies

## Abstract

**Background:**

Research has found that people with communication disabilities are three times more likely to encounter medical mishaps. Almost a third of patients with speech-language therapy (SLT) diagnoses have other medical conditions across more than one of the burden of disease categories. Fifty per cent of these patients present with communication disorders. Student healthcare curriculums focus on patient dynamics and field-specific diversities. It does not often include the skills and knowledge required to effectively communicate and treat those with communication disorders.

**Objectives:**

This study aims to describe the communication challenges and strategies employed by a group of final year Nursing, Medicine, Dietetics and Human Nutrition, Physiotherapy and Occupational Therapy students when managing adults with communication disorders.

**Method:**

A qualitative, phenomenological study design was used. Questionnaires were electronically distributed, and results were analysed thematically.

**Results:**

The most significant challenges whilst managing adults with communication disorders were patients’ receptive and expressive language difficulties. Further challenges included lack of knowledge surrounding communication disorders, lack of training in the use of appropriate communicative assistive devices, factors within the physical environment and gaps in students’ clinical performance. Strategies used to facilitate communication included caregiver assistance, gestures and written language.

**Conclusion:**

This study revealed that there is a need to develop healthcare students’ skills in managing adults with communication disorders. This is because of the challenges faced and inefficiency of the strategies used. Future research should focus on determining solutions for improved communication with adults with communication disorders. The study highlights the need for further education and training to address students’ communication needs with patients.

## Introduction

There are many risk factors contributing to communication disorders in adults living in South Africa. In a study conducted at a South African hospital, 77.48% of adults with speech-language therapy (SLT) diagnoses presented with non-communicable diseases, whilst 26.99% presented with communicable diseases. Of these 44.68% presented with speech disorders, 33.31% with language disorders and 10.95% presented with cognitive communication disorders. A total of 29.27% of these patients presented with multiple medical conditions across several burden of disease categories (Stone, Hoosen, Hochfelden, Maposa, & Singh, 2020).

According to Chichirez and Purcãrea (2018), communication is a clinical skill that is crucial in establishing a trusting relationship between the healthcare professional and the patient. Once trust is established, the patient becomes more comfortable in receiving services and disclosing personal information critical to the diagnosis (Ha & Longnecker, 2010). When the patient is confident that the healthcare provider has their best interest in mind, prognosis seems promising (Chichirez & Purcãrea, 2018).

Patients with communication disorders often cannot communicate effectively, thus creating a challenge for self-advocacy that impacts their quality of life (Stans, Dalemans, De Witte, & Beurskens, 2013). It is the responsibility of healthcare professionals to ensure that patient’s medical safety, such as correct diagnoses, well-communicated treatment plans, medication dosage information and follow-up appointments, is prioritised during service delivery (Vermeir et al., 2015). According to Murphy (2006), communication disorders cause a specific problem in primary care. This can compromise medical safety as it results in ineffective communication with healthcare professionals. This may lead to increased expenditure for unnecessary testing, the inappropriate prescription of medication, numerous referrals, which may result in increased cost as patients are required to attend more hospital appointments.

Nurses working with adult neurological patients reported feeling responsible for communication with patients. However, because of miscommunication, nurses often felt guilty of providing inadequate care as conversations were frustrating, leading to impatience and resulting in patients being dismissed (Hur & Kang, 2021). This highlights that miscommunication can lead to medical mishaps as the patient may not receive services in time to prevent their illness from deteriorating (Vermeir et al., 2015). These issues can be avoided with effective communication measures.

According to Stransky and Morris (2019), individuals with communication disorders experience challenges in interacting with their medical care team and difficulties in accessing medical care. Thus, adults report that their quality of health is reduced compared with others without communication disorders. Murphy (2006) found that patients encountered various challenges before consultations and when making bookings for medical appointments. These challenges included difficulty in using the phone, understanding the receptionist’s speech and the inability to write down an appointment date and time. During consultations, some patients required help from their carers whilst others felt disrespected when doctors addressed their caregivers instead of them. Some patients described unfair treatment from healthcare professionals, leading to misdiagnoses and poor implementation of treatment events (Sharby, Martire, & Iversen, 2015). General practitioners’ limited understanding of the nature and implications of communication disorders, results in mismanagement of adults with communication difficulties (Murphy, 2006).

A study conducted by Sharby et al. (2015) highlighted that environmental modifications enable persons with disabilities to participate in daily activities, which positively affect their quality of life. Factors in the physical environment such as lighting (type and position), acoustic environment, humidity and temperature, setting and furniture placement, written information and the availability of augmentative and alternative communication (AAC) systems (communication boards, manual signing eye gazing, etc.) is said to affect those with communication disorders and hinder their effectiveness in communication (Stans, Dalemans, De Witte, Smeets, & Beurskens, 2017). Morris, Dudgeon and Yorkston (2013) studied adults who use AAC and found that many barriers to receiving high-quality care existed, including difficulty in communicating with physicians, relationship building and establishing rapport. Stans et al. (2017) also identified written information and the availability of AAC as being a facilitator to improved understanding and communication between healthcare professionals and communication impaired patients.

Healthcare students are known to experience a great deal of stress during their training, including balancing their time to meet deadlines, studying for examinations and preparing for their practicals. As a result of these and many other factors, healthcare students may overlook the importance of effective communication with their patients (Teutsch, 2003). Although communication training is compulsory in most medical schools in South Africa and internationally, these usually focus on interpersonal skills and simplified communication in terms of medical jargon. However, a paucity of literature exists on whether this training includes skills focusing on communicating with those who have disabilities (Matthews & Van Wyk, 2018). For this reason, healthcare students may be unaware of the implications that miscommunication may have on treatment (O’Halloran, Hickson & Worrall, 2008).

Some challenges that healthcare professionals encounter whilst working with adults with communication disorders are because of their lack of knowledge about communication interactions (Burns, Baylor, Dudgeon, Starks, & Yorkston, 2015; Fox & Pring, 2005; Hemsley et al., 2008; Law et al., 2005; Morris et al., 2013; Murphy, 2006, as cited in Baylor, Burns, McDonough, Mach, & Yorkston, 2019). According to studies conducted both in South Africa and the United States of America, this lack of knowledge is a result of limited exposure and experience to communication disorders (Bastable & Dada, 2020; Sharby et al., 2015). Other challenges include lack of skills utilising AAC systems and communicating with those presenting with sensory impairments (O’Halloran et al., 2008).

McMillan et al. (2017) stated that to overcome the barriers and challenges that student healthcare professionals face, consultation with an SLT in order to develop skills and improve clinical practice with these patients is required. Shadowing of SLTs will improve skill development and collaborative interaction. These skills learnt as students will carry over to future interactions, resulting in better patient care. Successful intervention requires student healthcare professionals to consider and understand the patient, the multidisciplinary team and their caregivers. This includes the burden and stress of long-term care and life adjustments when caring for these individuals. Therefore, healthcare professionals should consider factors that might impact the emotional adjustment of caregivers (Toner & Shadden, 2002). Després (2017) identified strategies that student healthcare professionals can develop for effective communication to take place. These include being familiar, respectful, flexible and consistent with the patients’ preferred communication needs and methods. These recommended strategies could be taught to student healthcare professionals, thus allowing adequate time for their development in professional practice.

Stransky and Morris (2019) stated that general education on patient-provider communication is available; however, advanced training to address the needs of people with communication disorders is rare. Stransky and Morris (2019) stated that physicians rarely use supportive communication strategies that SLTs commonly train communication partners to use. This reflects a lack of exposure to these strategies. It was reported that doctors did not utilise strategies that patients found useful during communication. Strategies included writing down keywords whilst speaking, the use of visual aids to reinforce communication and the consistent use of meaningful gestures. These findings indicate a lack of healthcare professional awareness and adequate training on strategies to manage patients with communication disorders. This is further supported by student healthcare professionals reporting a lack of adequate training on how to interact with people with disabilities (Sharby et al., 2015), emphasising the need for further student healthcare training. In a study by Murphy (2006), it was found that general practitioners need information about communication strategies and practical tools to help improve medical consultations with individuals with communication disorders. Similarly, McMillan, Burrus, Willis and Grabowsky (2016) stressed the importance of teaching communication strategies to nursing students and highlighting its importance. Nurses reported a desire to learn communication methods and emphasised a need for support and education to facilitate communication. Nurses reflected that improved communication would ensure that patients are not excluded from benefits because of their communication impairments (Hur & Kang, 2021). Suggestions for how to teach communication strategies include instruction, role-playing activities or handouts. They also recommend professional terminology to be simplified to facilitate patient understanding. The SLTs can liaise with general practitioners to raise awareness of communication disorders and to facilitate improved communication (Murphy, 2006). A need to improve patient–provider communication in order to facilitate patient’s satisfaction and overall health outcomes is emphasised (Sharby et al., 2015).

According to Haidet et al. (2002), cultural differences between healthcare professionals and patients were identified as a barrier to communication. Student healthcare professionals’ awareness of this barrier can better prepare them to manage the cultural differences between themselves and their patients. To avoid negative attitudes and feelings towards healthcare professionals and the treatment they provide, it is important that student healthcare professionals have knowledge about the cultural and linguistic background of the various cultures in South Africa.

Stans et al. (2013) identified factors influencing effective communication in patients’ immediate social environment. These include efforts to improve communication, professionals’ knowledge, use of AAC devices, communication time constraints and the influence and power of the client. The study concluded that for effective communication healthcare professionals should develop better attitudes, awareness, knowledge and skills regarding their patients’ communication disorder. Further attention should be provided to adequately and effectively use AAC devices in clinical sessions to improve communication.

Ultimately the goal of healthcare provision is to improve the quality of life for those receiving it and miscommunication between healthcare professionals and patients may act as a barrier (O’Halloran et al., 2008). Furthermore, student healthcare professionals need to be mindful of the possible communication barriers between them and their patients. In addition, they require training of strategies to overcome these barriers to facilitate effective communication (Norouzinia, Aghabarari, Shiri, Karimi, & Samami 2016). Therefore, the purpose of this study was to describe what healthcare students identify as challenges and effective strategies when interacting with adults with communication disorders.

## Research methods and design

### Aim

The aim of the study was to describe the communication challenges and strategies of healthcare students whist managing adults with communication disorders.

### Research design

A qualitative, phenomenological research approach was suitable for the study in identifying the lived experiences and perspectives of student healthcare professionals (Edmonds & Kennedy, 2017; Mohajan, 2018). The phenomenon being studied was the communication experiences of student healthcare professionals when interacting with adults with communication disorders. The chosen design allowed the researchers to describe the communication challenges and strategies of healthcare students when interacting with adults with communication disorders.

### Setting

Participants were included from the School of Clinical Medicine, the School of Nursing and Public Health, Discipline of Dietetics and Human Nutrition and the School of Health Sciences (physiotherapy and occupational therapy [OT]) at a university in South Africa. As a result of the COVID-19 pandemic and lockdown restrictions, an electronic questionnaire was used.

### Study population

A purposeful sampling method was used as it allowed the researchers to deliberately select and identify healthcare students who were particularly experienced or had information about interactions with adults with communication disorders (Palinkas et al., 2015). The sample population consisted of 23 participants, which comprised medical (MBCHB), nursing, physiotherapy (PT), OT and dietetics and human nutrition students. The sample size fell within the recommended criteria for a phenomenological approach of 5–25 participants (Creswell, 2009).

Inclusion criteria required healthcare students to be in their final year of study and registered at this specific university in South Africa. Medical and allied healthcare students who would typically form part of the multidisciplinary team managing adults with communication disorders were included. Participants were included if they had experience in managing adults with communication disorders. The exclusion criteria were participants in their year of study other than their final year as their clinical exposure may not yet have included experience with adults with medical conditions resulting in communication disorders.

Participants were identified and recruited via the university notice system, student referrals and a link was emailed to the head of disciplines for circulation.

### Expert review

An expert review was conducted by a qualified SLT who provided his or her knowledge and expertise to the research study and approved its appropriacy and relevance (ASHA, n.d). The results of the expert review revealed that the data collection instrument and the aims and objectives were adequate to obtain information, which addressed the research question. Modifications were made to the length of the data collection instrument, and an estimated completion time was provided to participants, as recommended by the reviewer.

### Pilot study

This pilot study aimed to determine the effectiveness, credibility and appropriateness of the data collection tool developed by analysing the appropriateness of the results and its relevance to the proposed study. It also aimed to determine whether participants could understand and answer questions easily and accurately if the data collection tool was time effective and whether the layout, format and wording were appropriate. The pilot study followed the inclusion and exclusion criteria of the study to test the credibility of the data collection tool. An electronic questionnaire was administered and participants provided useful feedback about the readability of the data collection instrument and time taken to complete the questionnaire. Changes were made to the length and readability, by reducing redundancy and rephrasing questions, accordingly.

### Data collection

As a result of the COVID-19 pandemic, an electronic survey employing the use of a self-administered questionnaire was utilised, allowing better accessibility to all participants and to answer at their leisure without feeling overwhelmed, ensuring reliable results. The questionnaire included an informed consent page, allowing only those willing to participate to proceed. The participants were provided with a link to access the questionnaire, which was conducted via SurveyMonkey. Sixteen questions and 12 follow-up questions were included. Questions were primarily exploratory open-ended, some closed-ended and others follow-up questions requiring participants to elaborate on previous responses. The questionnaire concluded with questions directed at gaining possible solutions from the target population on the subject matter.

### Data analysis

Data were analysed using a thematic analysis with a general inductive approach to derive superordinate and subordinate themes. The first superordinate theme, communication challenges, included the following subordinate themes: (1) most common communication difficulties, (2) understanding the patient’s communication intent using nonverbal means, (3) physical environment and (4) the impact of communication disorders on providing an accurate diagnosis, treatment and patient compliance. The second superordinate theme, communication strategies, included the following subordinate themes: (1) implementation of communication strategies, (2) effectiveness of communication strategies implemented and (3) patient and caregiver involvement.

### Ethical considerations

Ethical clearance to conduct this study was obtained from the Biomedical Research Ethics Committee of University of KwaZulu-Natal (No. BREC/00001280/2020).

The questionnaire on SurveyMonkey commenced with an information sheet, which provided the nature and purpose of the study, together with an informed consent form. Once participants provided consent, they completed the survey.

## Results

A total of 23 final-year healthcare students over the age of 18 years, who were involved in the management team for adults with communication disorders, completed the questionnaire. Seven medical students, four nursing students, six physiotherapy students, two OT students and four dietetics and human nutrition students participated. The data collected from all participants were analysed and discussed according to superordinate and subordinate themes.

### Theme 1: Communication challenges

#### Sub-theme 1: Most common communication difficulties seen

Out of 23 healthcare students, 20 reported difficulty communicating with patients with communication disorders. Participants noticed challenges in their ability to obtain case history, understand the patient’s attempts to describe symptoms, provide instructions that would result in effective treatment and monitor progress. As reflected in Table 1, most participants found it difficult to communicate with patients and highlighted patients’ expressive language difficulties as most common, with some difficulties in patients’ receptive language. Out of the 23 participants, 7 allied healthcare students and 4 medical students indicated that difficulty in expressive language was most commonly seen.

**TABLE 1:** Participants’ responses on the most common communication difficulties seen.

Participant	Response on communication difficulties
PT 3	‘Sometimes I don’t understand what they are saying. I need to ask them again and again to actually get the word they are trying to pronounce.’
MBCHB 1	‘Taking history of illness from these patients is difficult. Taking consent from these patients is difficult, sometimes it is difficult to even assess their capacity to consent.’
MBCHB 6	‘Some patients have expressive aphasia so it’s difficult to get the relevant history and to elicit risk factors.’
PT 5	‘To conduct certain techniques, you need a patients’ co-operation and understanding, therefore the poor communication creates a barrier.’
MBCHB 6	‘It’s also hard to explain to them what the problem might be if they have receptive aphasia.’

MBCHB, medical; PT, physiotherapy.

#### Sub-theme 2: Understanding the patient’s communication intent using nonverbal means

Fourteen medical and allied healthcare students were able to, at times, understand patients’ communicative intent when expressing themselves non-verbally, with nine students reporting that they most often understood patients’ communicative intent. This included the use of gestures, eye gaze, vocalisations, etc:

‘It’s very much trial and error in addition to getting to know the client’s mannerisms and what their alternate methods of communicating are.’ (OT 1, August 2020)

‘I’ve used a pen and paper before to communicate with patients but I had no idea that if falls under the AAC. It’s my first time hearing about the AAC actually. I think they be very helpful, especially with patients who have motor aphasia. It gives the healthcare practitioner and the patient the ability to communicate and create rapport, thus working together for the patient’s rehabilitation which requires cooperation from the patient.’ (MBCHB 4, August 2020)

As reflected in Table 2, participants in the study reported feeling emotionally strained, frustrated and helpless when they struggled to understand a patient’s attempt to express their concerns using emotions, gestures and/or behaviours.

**TABLE 2:** Participants’ feelings related to difficulty understanding patients.

Participant	Response on feelings
DIETETICS 1	‘Very saddened as I want to do my job the best I can and make the patient feel comfortable I also feel irritated in myself that I cannot understand.’
MBCHB 4	‘It is frustrating! Especially as a student with little experience who’ll have to present that case to a consultant. You appear as if you’re lazy and are just making up excuses.’
OT 2	‘I feel bad that I cannot help the client and I can often see the client’s frustration with not being understood, which makes me feel terrible.’

OT, occupational therapy; MBCHB, medical.

The use of AAC systems during assessment and therapy sessions by patients, yielded different results for each healthcare discipline. Physiotherapy students mainly reported that their patients did not use AAC systems. All OT and dietetics and human nutrition students reported that their patients use AAC systems during the treatment sessions. Nursing students reported that their patients mostly use AAC systems. However, all medical students reported that their patients do not use AAC systems:

‘Makes communication less difficult as it provides extra means of communication style.’ (NURSING 3, August 2020)

Participants who reported that they are not trained to use AAC systems or devices were also asked if they would consider using these strategies. Participant responses indicated that some would consider using AAC:

‘I feel like with proper training of these methods it can help facilitate a more effective communication between the elderly and the nurse and attend the elderly’s needs.’ (NURSING 3, August 2020)

All participants in the study agreed that written information and the use of AAC facilitate communication. They further explained that it helps them understand the patient better and fosters a more effective two-way communication.

#### Sub-theme 3: Physical environment

Twelve healthcare students felt that the physical environment does not affect their communication with patients. However, as reflected in Table 3, some participants reported that it does affect communication. Healthcare students noticed that acoustic factors, such as a noisy or busy environment, lighting and temperature, impact effective communication.

**TABLE 3:** Factors in the physical environment that affect communication.

Participant	Response on environmental factors affecting communication
OT 2	‘The environment/setting definitely affects communication the most, if the environment around you is busy and noisy it will make it even more difficult to hear the client and it can be very distracting to both therapist and client. Also, the acoustic environment has a large affect.’
DIETETICS 1	‘Obviously the lighting in showing the visual aids or understanding the gestures, however I also find temperature an issue as patients irritability and my ability to stay “patient” is decreased.’
MBCHB 7	‘An environment that is too busy, noisy and chaotic makes it even more difficult to communicate with such patients.’

MBCHB, medical; OT, occupational therapy.

#### Sub-theme 4: Impact of the communication disorder on providing an accurate diagnosis, treatment and patient compliance

In this study, 20 of the 23 participants agreed that inadequate healthcare, incorrect diagnosis and inappropriate medication are provided when the patient has difficulty in communicating. As reflected in Table 4 most participants stated that if the patient could not communicate adequately, this may lead to misdiagnosis. Many also stated that healthcare is not the same when a communication barrier exists. A medical student further noticed that it can lead to misdiagnosis, undertreatment and overtreatment.

**TABLE 4:** Participants’ responses on the impact of communication difficulties on adequate service provision.

Participant	Responses on impact to adequate service provision
PT 6	‘Sometimes especially in public hospitals, a patient has minimum time with a healthcare individual so this can result in misdiagnosis when they find it hard to communicate.’
PT 6	‘Sometimes adults do not understand the treatment and no matter what technique is used they just cannot understand.’
OT 2	‘If the client cannot effectively communicate with his healthcare providers, there may be miscommunication or the client could be unable to communicate vital information that could result in misdiagnosis.’
DIETETICS 2	‘The nutrient intake may change as the dietitian may not have the full idea of the patients’ preferences of food or they have anorexia.’
MBCHB 3	‘If the patient has an underlying allergic reaction that he or she knows of and you might not obtain that information and go on to treat them with the drug, which they are allergic with which can cause dreadful consequences such as anaphylactic shock.’
MBCHB 7	‘I may not be aware of all of the patient’s ailments, resulting in some being unattended to.’

MBCHB, medical; PT, physiotherapy; OT, occupational therapy.

Participants who did not agree with the above were asked what in their communication efforts with patients, ensured that their goals were achieved. A physiotherapy student shared that they make use of manual techniques, which would provide indicators, and they would also make use of medical files or other members of a multidisciplinary team. A medical student shared that patients often come in with a family member and so they provide information that is required for further management of the patient. All medical students reported that their patients comply with treatment plans; additionally, eight allied healthcare students reported the same. In comparison, eight allied healthcare students shared that their patients do not comply with provided treatment plans:

‘At first presentation these patients come with a family member so we utilise them in acquiring necessary information that will guide further management of the pt.’ (MBCHB 5, August 2020)

‘I am in the physiotherapy field and we use manual techniques that would provide us with answers or indicators. When patients are unable to communicate during interview then we refer to the medical file or ask the other members of the MDT.’(PT 1, August 2020)

Healthcare students were asked whether their patients complied with treatment programmes or not. They were then asked to reason why. Healthcare students who said that their patients do comply with treatment programmes reasoned that the patients either know they are being provided with help or they want to be discharged, thus motivating them to comply with treatment programmes.

Those who shared that their patients did not comply with treatment programmes were also asked to reason why. As reflected in Table 5, participants shared that patients often lose hope, and thus intensive motivation is required; some patients had difficulty in understanding and being able to ask questions regarding the treatment plans.

**TABLE 5:** Participants’ responses on the effect of communication disorders on patient compliance to treatment programmes.

Participant	Responses on compliance to treatment programmes
OT 2	‘I think if they have trouble understanding the treatment plan you have provided there would be poorer compliance. Also, they may not be able to effectively ask questions they may have about the plan, which could result in incorrectly following the plan.’
DIETETICS 4	‘The patients are unable to vocalise how they are feeling and whether or not something makes them feel better or worse. Eventually, they just lose hope and give up. They require intense motivation.’
MBCHB 6	‘Patients often don’t get the optimum treatment as a patient who is able to express themselves and state what really the problem is. However, a physical examination has its role in making a diagnosis.’

OT, occupational therapy; MBCHB, medical.

Majority of the healthcare students agreed that gaps in clinical performance do serve as a barrier. Further participant elaboration observed that a lack of learning and full understanding impacts clinical performance and in turn communication:

‘I don’t know how to explain; however, lack of clinical performance prevents proper communication with the patient and thus not reaching a proper diagnosis early.’ (MBCHB 3, August 2020)

‘You can’t provide good service with gaps.’ (NURSING 1, August 2020)

### Theme 2: Communication Strategies

#### Sub-theme 1: Implementation of communication strategies

When communication difficulties arise, all nursing and dietetics and human nutrition students reported the implementation of communication-specific strategies. Most OT and physiotherapy students also implement strategies. In comparison, 4 of 7 medical students reported that they do not implement communication strategies. The most common strategies implemented across all disciplines were the use of gestures and writing down key information. Table 6 reflects the types of communication strategies used by participants when communicating with patients.

**TABLE 6:** Participants’ responses on the types of communication strategies used.

Participant	Responses on types of communication strategies
OT	‘Visual aids, gestures, body language, communication boards.’
PT 1	‘Break down what I am trying to communicate into the simplest form, if it’s a test or move I want them to do, I first do it so they can get the feel of what I want them to do.’
DIETETICS 4	‘The patient writes answers to my questions in a book. Patients with language barriers usually point to the vegetables that they do eat on a chart.’
MBCHB 2	‘We write words on a paper and ask the patient to nod for yes OR we get the patient to write what they want/their response.’
NURSING 3	‘Use not only hand gestures most of the time but also visual gestures but not writing down keywords because of lack of writing material in clinical settings.’

OT, occupational therapy; PT, physiotherapy; MBCHB, medical.

#### Sub-theme 2: Effectiveness of communication strategies implemented

Fifteen allied healthcare students and five medical students found that the communication strategies implemented were effective; however, some participants felt that their lack of training and limited resources hindered effective communication with patients:

‘With the communication barrier it’s difficult to gauge progress. I’m well aware that it’s my deficiency as a professional more than it is my client having a communication difficulty.’ (OT 1, August 2020)

‘The inability of the patients to express themselves, especially if they cannot write.’ (MBCHB 1, August 2020)

#### Sub-theme 3: Patient and caregiver involvement

Fourteen participants across all disciplines said that they requested caregiver assistance on occasion, with some of the nursing students reporting that they did not request caregiver assistance at all:

‘Sometimes the patient is confused, and we need confirmation from the caregiver.’ (DIETETICS 3, August 2020)

‘If the patient cannot verbalise properly about what happened. The caregiver provides a history. Also, in terms of taking consent. Nurses help if there is a language barrier.’ (MBCHB 2, August 2020)

Eleven healthcare students, including five allied and six medical students, in this study reported that they are not aware of how the patient feels when they request assistance from caregivers. The remaining participants are aware and thus adjust their approach to the patient and caregiver, ensuring patient inclusivity:

‘Yes I would always first ask the client and then would respectfully ask if the caregiver/nurse could add to the conversation but ensuring the client is involved and engaged as much as possible, also making sure the client agrees with what his caregiver/nurse is saying.’ (OT 2, August 2020)

‘I still communicate with the patient directly and ask the family member occasionally if I do not understand.’ (DIETETICS 1, August 2020)

Participants who were not aware of how the patient feels when they request assistance from caregivers have considered how it negatively impacts patient’s feelings and attitudes. One medical student said that they ‘have not thought of it’ and that their patients ‘have probably gotten used to it.’

‘Yes. It even affects me because the quality of healthcare provided to the patient is decreased and my goal is care to be patient-centred.’ (MBCHB 4, August 2020)

‘I have not thought of it, as some patients actually look at their caregiver to give the answer as they probably have gotten used to it.’ (MBCHB 3, August 2020)

## Discussion

### Theme 1: Challenges

This study found the presence of both expressive and receptive language disorders in patients to be the most common difficulty impacting communication with healthcare students. Likewise, Burns, Baylor, Dudgeon, Starks and Yorkston (2017) stated that qualified healthcare professionals experience difficulty whilst communicating during medical interactions with adults with communication disorders. McKnight et al. (2002) further reported that qualified nurses and physicians expressed problems obtaining patient-specific information, timeously, creating a challenge for patient self-advocacy, impacting quality of life (Stans et al., 2013).

Healthcare students’ lack of exposure to simple communication tools and a need for further education is evident in this study. These findings are similar to those by Murphy (2006), in which general practitioners required information about communication strategies and practical simple tools to help improve communication with patients.

According to McGilton et al. (2012), interacting with people who have communication disorders requires patience, sensitivity and understanding. As observed from the results in this study, healthcare students experience difficulty in understanding their patients’ concerns when expressed by gestures/behaviours and/or emotions. This highlights the need for all healthcare students, to have learning experiences with patients who have difficulty in communicating, in order to develop their skills and knowledge. However, this is rarely being done in learning and education environments.

The results in this study imply that allied healthcare students may have more knowledge and exposure to AAC systems compared with medical students. This may be because of the limited exposure to AAC and knowledge on how to check whether patients use AAC systems during their time of study. A study conducted by Sharby et al. (2015) found that individuals with disabilities feel that student healthcare professionals do not have the appropriate skills to effectively communicate with them. This is supported by Murphy (2006), who shared that general practitioner staff reported insufficient training in communication disabilities. Similarly, in this study, it was found that students in healthcare degrees reported a lack of sufficient training, leading to a feeling of less competence when working with patients with disabilities. This in turn can affect patient’s healthcare and healthcare experience.

Many healthcare students in this study reported a lack of knowledge about AAC. In agreement with these findings, McMillan et al. (2016) stated that to overcome the challenges that healthcare students face, improved clinical practice with patients and their SLT will provide more exposure to develop their skills.

Possible medical error and misdiagnosis because of communication disorders were found in this study. These findings are supported by a study by Després (2017) who observed that patients with communication disorders are at a higher risk of medical error because of the increase in communication breakdowns. Medical errors with patients can lead to misdiagnosis, serious injury to the patient and, in extreme cases, death (Després, 2017). Patients with communication disorders need safer communication environments that require training to develop skills in this area, which current healthcare professionals lack.

Patients’ noncompliance with medical plans is often attributed to a feeling of disrespect as seen in a study by Sharby et al. (2015), which included healthcare students. In contrast, the findings in this study indicated that many patients did comply with treatment plans. This could be attributed to the motivation provided by individual healthcare students.

### Theme 2: Strategies

Various communication strategies were successfully implemented by participants in this study. These findings are supported by those in a study by Baylor et al. (2019), who reported that patients who have communication disorders benefit when student healthcare professionals use multimodal communication such as gestures, writing and pictures. Diehl (2016), who found that the use of multimodal communication with patients reduces communication breakdowns, thus improving patient’s understanding and ability to express themselves lends further evidence in this regard.

Lack of adequate participant training in this study influenced the effectiveness of communication strategies used with patients. This barrier in communication can be attributed to the healthcare professional’s lack in patient–provider communication skills whereby information provision to the patient is inadequate further impairing patient understanding (Baylor et al., 2019). Therefore, miscommunication contributes to poor service delivery and ineffective treatment.

Common situations that led participants in this study to request assistance from caregivers included inability to obtain case history information from the patient, confused patients, if the patient had difficulties understanding instructions, was unresponsive or hard of hearing. Similarly, findings in a study by Baylor et al. (2019) found that factors contributing to student healthcare professionals request for assistance from caregivers were communication difficulties because of aphasia and dysarthria. In support of this study’s findings, Burns et al. (2015) also observed that patients and caregivers often worked together to ensure communication success when interacting with physicians when communication breakdowns occur. However, caregivers in this study felt that physicians should direct their questions to their patients instead of directly at the caregivers as a sign of respect. Similar findings were noticed by Burns et al. (2015).

Burns et al. (2015), stated that although physicians want to try to adjust their communication approaches, they may not know how to. This was evident from a medical student’s response in this study, which reflected that their lack in communication skills affects the quality of services they provided.

## Conclusion

The aim of the study was to describe the challenges experienced and strategies utilised by healthcare students whilst managing adults with communication disorders. Their lack in expertise and communicative flexibility often leads to healthcare students being overwhelmed and anxious, affecting the quality of healthcare provided. Various challenges existed including lack of knowledge surrounding communication disorders, training in the use of appropriate communication assistive devices, acoustic and visual factors within the physical environment. Strategies utilised to facilitate communication predominantly included writing down key information, using gestures and caregiver assistance. A study limitation is that participants included healthcare students from only one university in the country, and therefore the results of the study cannot be generalised to a wider body of healthcare students. The study indicates that whilst students studying at this university employ several strategies when challenges are encountered, there is still a need to further develop their skills and training when managing adults with communication disorders.
